# "Frost-Bite" in the Trenches

**Published:** 1915-11-06

**Authors:** C. R. Rutland

**Affiliations:** 83a Chester Square, S.W.


*To the Editor of* THE HOSPITAL.

### "Frost Bite" in the Trenches.

*To the Editor of* THE HOSPITAL.

SIR,—It is generally admitted that this condition, which proved so serious a source of suffering and loss amongst our troops last winter, was due to the defective leg-wear provided by the authorities. The puttee, which is in many respects an admirable covering, being warm, elastic, fairly permeable, and a good protective against cold, damp, thorns, branches, and dense undergrowth, has the fatal defect due to the means of fastening, which consists of a long inelastic tape which must be wound tightly round the leg just below the knee, thus interfering with the blood circulation, especially when the material shrinks under the influence of moisture inevitable in the trenches in winter.

Early in the spring of the present year the *Lancet* and the *British Medical Journal* called attention to this matter, and invited their readers to suggest a fastening that would be free from the defects of the tape. I devised a very simple "hook-pin" and submitted it to the Editor of the *British Medical Journal,* who wrote in the issue of June 12 : "It appears not only completely to answer the purpose in respect of puttees, but is likely also to replace the safety-pin for fixing bandages."

If this device is to prove of real value to our troops and to our wounded soldiers in the hospitals, it is essential that it should be tested as widely and thoroughly as possible, and at once. I have had a number of these hook-pins made, and will gladly send a specimen to any wearer of puttees, or to any nurse or surgeon, or to anyone interested who has a friend acting in either capacity.—I am, yours obediently, C. R. RUTLAND, M.D.

83A Chester Square, S.W., November 3, 1915.

